# AI Era Is Coming: The Implementation of AI Medical Devices to Endoscopy

**DOI:** 10.31662/jmaj.2024-0205

**Published:** 2024-09-27

**Authors:** Yosuke Tsuji, Mitsuhiro Fujishiro

**Affiliations:** 1 Next-Generation Endoscopic Computer Vision, Graduate School of Medicine, the University of Tokyo, Tokyo, Japan; 2 Department of Gastroenterology, Graduate School of Medicine, the University of Tokyo, Tokyo, Japan

**Keywords:** artificial intelligence, endoscopy, implementation

In recent years, artificial intelligence (AI) has made remarkable progress and is beginning to be introduced into various medical fields. In a review article entitled “Implementation of artificial intelligence in colonoscopy practice in Japan,” Misawa et al. outlined the following: 1) for what kind of aims is AI used in colonoscopy; 2) the path to regulatory approval and reimbursement of the AI medical devices they were involved in; and 3) the future challenges for colonoscopy using AI ^(1)^. “EndoBRAIN-EYE,” in which they were involved, is a successful example of an AI medical device with proven clinical efficacy, regulatory approval, and reimbursement. As one of the leading countries in digestive endoscopy, Japan has developed many endoscopy-related AI devices, and it is expected that many more products will follow in the future and Japan will lead the world in this field.

Although it is important to demonstrate clinical efficacy of AI devices, it is unexpectedly difficult to show their usefulness. In the field of colonoscopy, one important factor for clinical usefulness of AI is that “AI can detect more adenomas.” Many randomized controlled trials (RCTs) have shown this effect. However, it should be noted that RCTs differ from real-world clinical settings. In a study by Nehme et al, when AI was introduced in clinical practice and the decision to use it was left to the clinician, the actual rate of AI use was only 52%. Moreover, as a result, adenoma detection was not improved after the implementation of the AI device ^(2)^. Many of the physicians who participated in the study rated the AI as being distracting with many false positives. When we think of RCT to demonstrate the efficacy of AI-assisted endoscopy, there are some hurdles: 1) it is impossible to make RCT a blinded one; 2) a tandem trial design cannot be helped (“tandem” trial means conducting endoscopy twice in a row for the same patient); and 3) endoscopists are inclined to observe with more enthusiasm than usual because they are inevitably concerned about AI. The implementation study like the one by Nehme et al. is another option, but not a perfect one. As Misawa et al. pointed out, the usefulness of AI must continue to be examined, even after reimbursement is obtained.

Conversely, in 2018, Hirasawa et al. reported the world's first AI for gastric cancer detection. Afterwards, many AI devices assisting upper gastrointestinal (GI) endoscopy have been reported from Japan ^(3), (4)^. In upper GI endoscopy, it is more difficult to pick out lesions than in colonoscopy because early gastric or esophageal cancer is often not protruded but depressed or flat, so clinical applications of AI medical devices in this field are greatly anticipated. Compared to colonoscopy-related AI, there have been less reports concerning the efficacy of AI assisting upper GI endoscopy at the present time. In order to obtain regulatory approval and reimbursement also in AI devices focusing on upper GI endoscopy, we need to publish more evidence. Moreover, as Misawa et al. pointed out in their article, we need to present cost-effectiveness before acquiring reimbursement. A cost-effectiveness analysis regarding a computer-assisted diagnosis support system (CADx) for early gastric cancer in patients with moderate to severe atrophic gastritis due to *Helicobacter pylori* infection showed that an incremental cost-effectiveness ratio of less than 50,000 USD/QALY was achieved if the cost of CADx was less than 104 USD ^(5)^. The accumulation of such studies would be of help for obtaining health insurance coverage.

How to use AI in a real clinical setting is quite an important issue. Especially, “who is finally responsible for a clinical result?” should be well considered before the implementation of AI. As of now, AI is not God; it cannot avoid false-positive or false-negative diagnosis. Considering that, human doctors must take responsibility for the final clinical decision. Japan Gastroenterological Endoscopy Society provides guidelines for the clinical use of AI software (https://www.jges.net/wp-content/uploads/2023/05/AIsoft.pdf). The guidelines clearly state that physicians are responsible for diagnosis and that AI should be used only adjunctively. In other words, we should make good use of AI in various clinical settings and contribute to better health of many people around the world.

In conclusion, Japan, which has led the world in the field of GI endoscopy, must demonstrate the clinical and economic benefits of AI to support GI endoscopic diagnosis, lead the world in social implementation, and build further evidence.

## Article Information

### Conflicts of Interest

Next-Generation Endoscopic Computer Vision is an endowment department, supported with an unrestricted grant from AI Medical Service Inc.

### Author Contributions

The first draft of the manuscript was written by Yosuke Tsuji and Mitsuhiro Fujishiro commented on the draft versions of the manuscript. Both authors read and approved the final manuscript.
